# A specific role for septohippocampal acetylcholine in memory?

**DOI:** 10.1016/j.neuropsychologia.2012.07.022

**Published:** 2012-11

**Authors:** Alexander Easton, Vincent Douchamps, Madeline Eacott, Colin Lever

**Affiliations:** Department of Psychology, University of Durham, Durham, DH1 3LE, UK

**Keywords:** Oscillations, Theta, Scopolamine, Rats, Episodic memory, Spatial memory

## Abstract

Acetylcholine has long been implicated in memory, including hippocampal-dependent memory, but the specific role for this neurotransmitter is difficult to identify in human neuropsychology. Here, we review the evidence for a mechanistic model of acetylcholine function within the hippocampus and consider its explanatory power for interpreting effects resulting from both pharmacological anticholinergic manipulations and lesions of the cholinergic input to the hippocampus in animals. We argue that these effects indicate that acetylcholine is necessary for some, but not all, hippocampal-dependent processes. We review recent evidence from lesion, pharmacological and electrophysiological studies to support the view that a primary function of septohippocampal acetylcholine is to reduce interference in the learning process by adaptively timing and separating encoding and retrieval processes. We reinterpret cholinergic-lesion based deficits according to this view and propose that acetylcholine reduces the interference elicited by the movement of salient locations between events.

## Highlights

► We review effects of lesion/pharmacological disruption to the cholinergic system. ► We review a model of the role of septohippocampal acetylcholine in memory. ► We review recent behavioural loss-of-function and electrophysiological data. ► We use the model to explain deficits after septohippocampal cholinergic disruption.

## 1 Introduction

Acetylcholine (ACh) has long been implicated as a neurotransmitter in learning and memory (e.g., Drachman, 1977). It projects widely throughout the central nervous system (Mesulam, Mufson, Wainer, & Levey, 1983) but the cholinergic projections from the basal forebrain to the cortex and hippocampus contained within the medial septum and vertical limb of the diagonal band (MS/VDB), in particular, have been linked to memory functions (e.g., Easton, Ridley, Baker, & Gaffan, 2002; Hasselmo, 2006; Ridley, Barefoot, Maclean, Pugh & Baker, 1999). Lesions of the basal forebrain affecting these cholinergic projections can result in profound amnesia in humans (see Deluca & Diamond, 1995 for review; Norlen & Olivecrona, 1953). Although these findings support the view that structures in the region of the basal forebrain are necessary for memory, the damage is rarely limited to the regions of cholinergic projections, and is certainly not selective for ACh, as the region contains cells other than those that express ACh. Therefore, the link between memory and ACh in these patients is circumstantial.

Perhaps more indicative of the role of ACh is Alzheimer’s disease, although this disease involves a general loss of cognitive function and not just a loss of memory. However, impaired memory (and in particular, impaired episodic memory) is apparent early in the disease (e.g., Collie & Maruff, 2000) and the severity of the memory impairment is correlated with the degree of cholinergic loss in these early stages (Bierer et al., 1995). As a result, much research has been concentrated on the effects of cholinergic enhancement on memory performance in patients with Alzheimer’s disease (for review see Popp & Arlt, 2011). Nonetheless, any memory impairment in Alzheimer’s disease co-occurs with a variety of other pathological changes, making it difficult to identify the specific role of ACh. Therefore, in order to fully understand the role of this neurotransmitter system it is helpful to consider those manipulations that specifically alter cholinergic function.

### 1.1 Selective lesions of acetylcholine in primates

If the cholinergic projections to cortex and hippocampus are important in memory, disruption to these projections should result in severe memory impairments, even in the absence of direct damage to the cortical regions. Horel (1978) proposed that white matter damage in the temporal cortex in patients such as H.M. (Scoville & Milner, 1957) would disconnect these cholinergic projections and that this might contribute to the dense amnesia in such patients. Certainly H.M. is seen to have substantial damage to the amygdala and the white matter of the anterior temporal stem bilaterally (see Fig. 2, panels H–J Corkin, Amaral, Gonzalez, Johnson, & Hyman, 1997), and it is via both these routes that cholinergic cells of the basal forebrain project to regions of the inferior temporal cortex (Mesulam, 1995). In turn, regions of the inferior temporal cortex are known to be important for memory, for example lesions to this region causing impairments in object recognition tasks (e.g., Buckley & Gaffan, 1997; Eacott, Gaffan, & Murray, 1994; Eacott & Heywood, 1995; Murray, 2005), and it seems possible that a combination of cholinergic deafferentation of these regions along with direct damage to the hippocampus could severely disrupt a range of different memory types. To test this hypothesis directly, Gaffan, Parker, and Easton (2001) sectioned the white matter of the anterior temporal stem and the amygdala bilaterally, in addition to sectioning the fornix (the route of subcortical communication with the hippocampus, including cholinergic connections) in monkeys. This white matter lesion produced a very severe anterograde memory impairment, with impairments in visual discrimination, recognition memory and memory for scenes which are considered analogous to episodic memory (Gaffan, 1994). In contrast, there was an absence of retrograde amnesia, with memory for similar problems learnt prior to the surgery being preserved (Gaffan et al., 2001; Gaffan, Easton, & Parker, 2002). Gaffan argued therefore, that this modelled anterograde amnesia, although retrograde amnesia may have its basis in additional damage.

Although this large white matter lesion, including disconnection of the hippocampus and inferior temporal cortex from their cholinergic inputs resulted in a substantial anterograde amnesia, the specific role of ACh was not demonstrated through these experiments. However, more specific lesions of cholinergic cells can be made using an immunotoxin. By injecting an immunotoxin into the basal forebrain, cholinergic projections to cortex and hippocampus can be destroyed whilst other non-cholinergic projections from the basal forebrain remain intact (Wiley, Oeltmann, & Lappi, 1991). Injecting an immunotoxin into the basal forebrain in one hemisphere in combination with an ipsilateral fornix lesion isolates the hippocampus from ACh projections unilaterally. When such a lesion was combined with a lesion of the inferior temporal cortex in the opposite hemisphere preventing visual input to the hippocampus in the other hemisphere, Easton et al. (2002) showed that an anterograde amnesia resulted which was comparable to that following the substantial white matter lesion of Gaffan et al. (2001). Thus, the specific role of ACh in this task could be identified. This finding supported earlier studies in marmosets (Ridley, Pugh, Maclean, & Baker, 1999) where immunotoxic lesions of the basal forebrain were also seen to produce specific memory impairments.

However, in these studies all the cholinergic cells of the basal forebrain are lesioned, and it is possible that some of the memory impairment results from disruption of cholinergic projections other than to the specific target area. Recent studies in primates, therefore, have targeted immunotoxic lesions of cholinergic projections into the target region only. As the immunotoxin used can be retrogradely transported by the cells, injecting the immunotoxin into, for example, the rhinal cortex serves to deplete that region alone of cholinergic input whilst other input remains intact, as do cholinergic projections to other brain regions. Nonetheless, Turchi, Saunders, and Mishkin (2005) demonstrated that such immunotoxic lesions of the rhinal cortex produce substantial memory impairments, in this case of recognition memory (delayed non-match to sample; DNMS), further supporting a role for ACh in some forms of memory. In contrast, however, Browning, Gaffan, Croxson, and Baxter (2009) demonstrated that cholinergic lesions of the inferior temporal cortex do not impair recognition memory or the episodic-like scene learning impaired in the study by Easton et al. (2002) although both tasks are impaired by complete lesions of this cortical region (Buckley, Gaffan, & Murray, 1997; Eacott et al., 1994; Easton & Gaffan, 2000). The same animals are impaired at the scene learning task following a subsequent fornix lesion (Browning et al., 2009) and this impairment is more severe than the simple additive effects of either lesion on their own. DNMS remained unimpaired following the addition of the fornix lesion (Browning et al., 2009).

The data from monkeys, therefore, shows that the cholinergic system is important for normal memory, especially of an episodic memory analogue (Browning et al., 2009; Easton et al., 2002), although there is conflicting evidence for its role in recognition memory (Browning et al., 2009; Turchi et al., 2005). In addition, it appears that there may be an interplay between cholinergic deafferentation of the inferior temporal cortex and the addition of fornix lesions. However, it cannot be ruled out that the addition of the fornix lesion in Browning et al. (2009) study merely reflects additional cholinergic deafferentation of medial temporal lobe memory systems. If ACh is important for memory within cortex and the hippocampus, then we need to better understand its specific function.

## 2 What are the key functions of acetylcholine in memory?

Acetylcholine is believed to control a repertoire of responses to novel information. As we set out below, this includes increasing exploratory behaviours directed towards sources of novelty (Lever, Burton, & O’Keefe, 2006; Thiel, Huston, & Schwarting, 1998), and enhancing synaptic plasticity for encoding novel associations (e.g., Drever, Riedel, & Platt, 2011; Segal & Auerbach, 1997; Sugisaki, Fukushima, Tsukada, & Aihara, 2011). A third cholinergic function, which this review focuses on, would consist of promoting efficient learning of novel information by preventing the retrieval of previously-existing associations interfering with the new encoding (proactive interference). In order to do so, encoding and retrieval states need to be separated within memory systems. Similar interference-related problems encountered in computational modelling of associative encoding and retrieval have encouraged a set of models by Hasselmo, Bodelón, and Wyble (2002), Hasselmo, Wyble, and Wallenstein (1996), Kunec, Hasselmo, and Kopell (2005), Meeter, Murre, and Talamini (2004) which formally describe how this might be achieved.

### 2.1 The encoding versus retrieval scheduling (ERS) framework

The ‘encoding versus retrieval scheduling (ERS)’ framework refers to the set of models that deal with the problem of how the hippocampus separates and schedules encoding and retrieval. There are two approaches to the problem within the ERS, acting at different timescales. We will argue here that they are complementary. The first involves levels of hippocampal ACh, which when high support encoding and inhibit recurrent networks subserving pattern completion-based retrieval (Hasselmo, 1999; Hasselmo, 2006; Hasselmo & Schnell, 1994a; Hasselmo et al., 1996; Meeter et al., 2004). This cholinergic influence on the ERS dynamics would last seconds to minutes. The second approach involves the hippocampal theta rhythm, a 4–12 Hz oscillation, under which time-windows for encoding and retrieval wax and wane several times a second, each preferentially occurring at a different theta phase (i.e., at a different time relative to the oscillation; Hasselmo et al., 2002). We introduce and summarise recent data supporting the ERS framework, including our findings from recordings of pyramidal cells in hippocampal region CA1 in freely behaving rats under manipulations of novelty and cholinergic antagonism. Finally, we use the ERS framework to guide our interpretation of the deficits that result from lesions of cholinergic septohippocampal neurons.

Our recent findings (Douchamps, Jeewajee, Blundell, Burgess, & Lever, 2011; Lever et al., 2010) relevant to this model are summarised briefly here before a more detailed presentation below, in order to motivate our presentation of the ERS framework. We show that in environmental novelty, a situation in which hippocampal ACh is high and encoding is prioritised, CA1 pyramidal cells fire at a later phase of the theta oscillation than they do when an environmental context is familiar, an effect termed the ‘later-theta-phase-in-novelty effect’. Thus, as envisaged by the ERS framework, encoding and retrieval preferentially take place at different phases of hippocampal theta. Second, systemic scopolamine (a cholinergic muscarinic antagonist with amnestic, ‘anti-encoding’ properties) induces an earlier theta phase of firing when given in a familiar environment. Third, when systemic scopolamine is given during environmental novelty, the ‘later-theta-phase-in-novelty effect’ is abolished (i.e., firing occurred at a similar phase to, or earlier than, in the familiar environment. In combination, these effects strongly suggest that both ACh and theta are involved in the hippocampal scheduling of encoding and retrieval. Therefore, our findings appear to support a combination of both approaches in the ERS framework.

As we discuss below, the hippocampal memory system should ideally: (1) prioritise encoding of novel information when situations are novel/unexpected; (2) not be incapacitated by interference when a cue has more than one association over a given period. We consider each of these two desirable properties in turn.

### 2.2 The hippocampus and neuromodulation: Acetylcholine levels increase in novelty

It is clearly adaptive for an efficient memory system to set up models of the external world based on past experience and to update these models both periodically and, crucially, when their predictions fail (e.g., Lever et al., 2006; O’Keefe & Nadel, 1978). In contrast, comprehensively encoding model-confirmations is less important. Therefore, it is likely a general feature of such an efficient system that encoding (model updating) should be prioritised when contexts and elements of contexts are novel and/or unexpected. A reliable finding that in novel situations hippocampal levels of ACh increase (Giovannini et al., 2001; Thiel et al., 1998; is consistent with such a system. For example, there is evidence of neuromodulatory feedback loops whereby the hippocampus can initiate a novelty-dependent process that ultimately modifies its own function. Specifically, the hippocampus detects novelty and sends a novelty signal to downstream targets, which ultimately cause the subcortical neurons in question (medial septum/DBB) to release the neuromodulator into the hippocampus itself (Hasselmo & Schnell, 1994; Hasselmo & Wyble, 1997; Lisman & Grace, 2005; Meeter et al., 2004). The levels of the neuromodulator in the hippocampus reflect the degree of novelty detected and thereby control its memory function. In this review, we will pass over suggested novelty detection mechanisms and signal transfer stages, and focus on the latter part of the process, in which levels of ACh in the hippocampus have increased and set the dynamics which bias the system towards successful encoding.

### 2.3 Acetylcholine enhances novelty-responsiveness: Exploration, plasticity and the encoding mode

Acetylcholine enhances at least three functions useful in novelty: exploration, long-term synaptic plasticity, and the encoding mode (i.e., prioritisation and encoding of novel information). Clearly, encoding a new environmental context and its contents, or updating an existing model of a changed context, is more adaptive if the context is sampled from multiple viewpoints. It is increasingly clear that the hippocampus controls exploration that aids this encoding (e.g., Lever et al., 2006; O’Keefe & Nadel, 1978; Saab et al., 2009; Voss, Gonsalves, Federmeier, Tranel, & Cohen, 2011). In rodents, for example, rearing on the hind legs increases in novel environments (e.g., Anderson et al., 2006; Hunsaker, Rosenberg, & Kesner, 2008; Lever et al., 2006; Wells, Krikke, Saunders, Whittington, & Lever, 2009), increasing the number of viewpoints sampled, and is clearly a novelty-responsive exploratory behaviour that is strongly modulated by the hippocampus and hippocampal ACh (reviewed in Lever et al., 2006). For instance, increases in hippocampal ACh levels (in CA1) on first exposure to a novel environment correlate very strongly with the initial levels of rearing in that novel environment (Thiel et al., 1998). Similarly, infusing muscarinic agonists directly into the hippocampus (dentate gyrus) greatly increases rearing in a novel environment (Flicker & Geyer, 1982). Acetylcholine, therefore, enhances the exploration that clarifies what new information should be encoded.

There is also clear evidence of enhancement of long-term plasticity by ACh (Blitzer, Gil, & Landau, 1990; Drever et al., 2011; Segal & Auerbach, 1997). For example, in spike-timing dependent plasticity protocols, ACh widens the temporal window within which long-term potentiation can be achieved. Specifically, *post*synaptic-before-*pre*synaptic activation sequences (e.g., 20 ms post-pre interval) which normally elicit long-term depression, under the influence of high ACh levels elicit long-term potentiation (LTP) (Sugisaki, Fukushima, Tsukada, & Aihara, 2011b; Zhang, Lau, & Bi, 2009). Acetylcholine also reduces the induction threshold for LTP in other LTP-inducing protocols (Seol et al., 2007; Sugisaki et al., 2011). Therefore, ACh clearly modulates plasticity.

We now turn to ACh’s control over the state of encoding. The first solution proposed by Hasselmo and others to the ERS problem in the hippocampus focused on the idea that Ach controls the balance between encoding new information and retrieving previous representations (Hasselmo & Schnell, 1994; Hasselmo, Wyble & Wallenstein 1996; Meeter et al., 2004). We describe the mechanism of this control below. We first need to consider two types of inputs to hippocampal region CA1.

A useful simplification is that CA1 has two input streams. The main contribution of one input stream (from the entorhinal cortex, layer 3) is to provide accurate information about the current sensory environment. The main contribution of the other input stream (CA3) is to make predictive inferences based on past associations (associative recall). Hasselmo and colleagues propose that ACh controls the relative influence upon CA1 of the excitatory sensory afferent input stream from the entorhinal cortex, versus the excitatory recurrent input stream from CA3 subserving pattern completion (Hasselmo & Schnell, 1994; Hasselmo & Wyble, 1997). Pattern completion is the process by which the inputs associated with only a partial set of cues are able to trigger retrieval of the entire (or more complete) representation originally associated with the complete set of cues. For example, it is adaptive to recall the location of food one has buried in a summer when one returns there in winter when some of the cues to food location, such as the position of flowers, are no longer available. Pattern completion following attractor dynamics has now been demonstrated in hippocampal spatial representations (Wills, Lever, Cacucci, Burgess, & O’Keefe, 2005), with good evidence that CA3 plays a crucial role (Nakashiba, Young, McHugh, Buhl, & Tonegawa, 2008; Nakazawa et al., 2003), as early models had proposed (Marr, 1971; Mcnaughton & Morris, 1987; Treves & Rolls, 1994). A key point of the ERS framework is that it is adaptive for the pattern completion retrieval process to be inhibited during encoding.

**Fig. 1:**
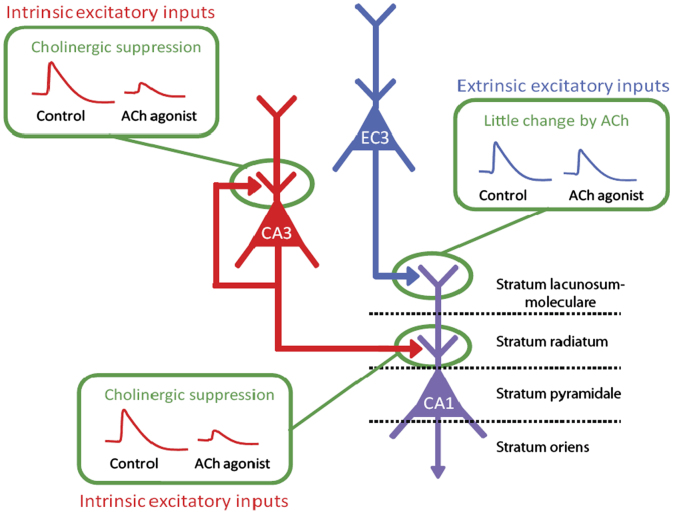
Selective suppression of intrinsic hippocampal connectivity by acetylcholine. ACh has different effects upon the two main excitatory inputs to CA1: entorhinal cortex layer 3 (extrinsic input) and CA3 (intrinsic input). ACh strongly presynaptically inhibits synaptic transmission in CA1 stratum radiatum (CA3 to CA1 synapses), and CA3 recurrent collaterals (CA3–CA3 synapses), while relatively sparing transmission in CA1 lacunosum-moleculare layer (entorhinal–CA1 synapses). Thus, acetylcholine protects to-be-encoded input patterns from the proactive interference arising from read-out of CA3. Based on data reviewed in Hasselmo (2012).

The role of ACh in the prioritisation of encoding would depends on its strong presynaptic inhibition of the excitatory recurrent feedback inputs, while mildly or not affecting excitatory sensory afferent input (reviewed in Hasselmo (2012)). Fig. 1 summarises ACh’s region-specific effects in the hippocampus, focusing on the inputs into CA1. Acetylcholine produces strong presynaptic inhibition of CA3–CA3 excitatory recurrent collateral synapses and the CA3–CA1 excitatory Schaffer collateral synapses in stratum radiatum, while only mildly affecting the layer 3 entorhinal-cortex–CA1 synapses in stratum lacunosum-moleculare. Simply put, ACh only mildly affects the input stream carrying information about current sensory cues (entorhinal cortex), while inhibiting both elements of the pattern completive retrieval input stream (CA3–CA3, CA3–CA1). Why is this input modulation useful?

### 2.4 Suppressing recurrent inputs during encoding reduces interference

The point of suppressing the recurrent inputs during encoding is to prevent interference; specifically, to prevent retrieval of previously encoded associations during the encoding of new associations. Hasselmo illustrates this using a simple CA3-type network model of a common version of the paired associates task, where words are common to both the first and second list of word pairs (for detailed presentation, see Hasselmo, 2012 pp. 187–193). Briefly, there is no difficulty with word pairs on the first list, such as a pairing of ‘Leather’ and ‘Holster’, for example, presented in the first context. The problem comes with the second list when ‘Leather’ is now paired with a new associate ‘Boot’. The essence of the problem is the Hebbian co-activity based strengthening, at encoding, of the synapse between the neuron representing the second context and the neuron representing ‘Holster’, which was active due to the retrieval of the previous ‘Leather’ and ‘Holster’ association. Later presentation of the cue word ‘Leather’ may now elicit the incorrect response ‘Holster’, or elicit response competition with the correct response, ‘Boot’. The solution is to prevent the inappropriate re-activation of the ‘Holster’ neuron during the List 2-encoding stage by inhibiting the recurrent synapse from the ‘Leather’ to ‘Holster’ neuron at that stage. Under this learning rule, later presentation of the cue word ‘Leather’ correctly elicits the ‘Boot’ response. Crucially, it is high ACh that instantiates the rule and inhibits the ‘Leather’ to ‘Holster’ synapse when the system is encoding the ‘Leather-Boot’ and ‘Context 2-boot’ associations.

### 2.5 Acetylcholine reduces interference resulting from previous associations

Therefore, ACh will be particularly useful in reducing interference that results from a cue having more than one association over the course of a task. The ERS framework predicts that the effect of cholinergic disruption would be to impair performance in such tasks, but predicts no particular advantage of cholinergic signalling in tasks where a given cue has a single stable association. The ERS framework was indeed used by Hasselmo, Stern and colleagues to successfully predict that scopolamine would more strongly impair encoding on the version of the paired-associate task where words appeared on both lists than on the version where words appeared on one list only (Atri et al., 2004).

This point is underlined by looking at a completely different task, the Morris water maze used with rodents. The ERS framework predicts no particular advantage from ACh in a version of the task when the goal location is stable across days, but predicts a cholinergic advantage in a version of the task where the location predicting the goal platform changes regularly, such that today’s goal location was a non-goal location yesterday, and vice versa. As discussed in detail below, we interpret the deficit in the cholinergic lesioned rats in Baxter, Bucci, Wiley, Gorman, and Gallagher (1995) water maze study in exactly these terms: the cholinergic-lesioned rats are impaired when the goal location changes from day to day.

### 2.6 Low levels of acetylcholine aid consolidation and retrieval

The complement of high levels of ACh improving encoding in the ERS framework of that low levels of ACh aid consolidation and retrieval (e.g., reviewed in Hasselmo, 2012). Acetylcholine levels are low in the two ‘offline’ states of quiet rest and slow wave sleep, and both these global states are associated with sharp wave/ripple oscillations in which memory consolidation occurs along the pathways identified with retrieval, notably including CA3–CA3, CA3–CA1, CA1–entorhinal synapses. (O’Neill, Pleydell-Bouverie, Dupret, & Csicsvari, 2010; Sutherland & McNaughton, 2000) provide useful reviews.

### 2.7 Interim summary of ERS framework regarding the role of acetylcholine in memory operations

Overall, therefore the ERS framework predicts for encoding an advantage from *high* ACh and a disadvantage from anticholinergic disruption. For retrieval and consolidation it predicts an advantage from *low* ACh and an advantage from anticholinergic disruption. When a cue takes on more than one association during the course of a task, the advantage from the high ACh levels at encoding will be most pronounced. Recent comprehensive reviews of at least *pharmacological* manipulation of cholinergic transmission suggest that overall the predictions of the ERS framework are confirmed (Bentley, Driver, & Dolan, 2011; Micheau & Marighetto, 2011). These include but extend well beyond demonstrations of impairments in encoding from anti-cholinergic disruption, although as later discussed, the evidence from lesions of cholinergic septohippocampal neurons suggests a clearly narrower range of impairments that deserves scrutiny. Here, we will focus on the specific disruption to encoding in hippocampally dependent tasks by scopolamine, a non-specific muscarinic antagonist.

### 2.8 Scopolamine impairs hippocampal-dependent memory, and may particularly impair encoding

Scopolamine is so well-established as an amnestic drug that it is often used as a model to induce a memory deficit which then a potential promnestic agent may redress (e.g., Norman, Brooks, Hennebry, Eacott, & Little, 2002). In rats, systemic or central administration of this drug impairs performance in several hippocampus-dependent tasks including the Morris water maze (Herrera-Morales, Mar, Serrano, & Bermudez-Rattoni, 2007; Janas et al., 2005; von Linstow Roloff, Harbaran, Micheau, Platt, & Riedel, 2007), the radial arm maze (Masuoka, Fujii, & Kamei, 2006; Mishima et al., 2000), spatial alternation in a T-maze (Givens & Olton, 1995), contextual fear conditioning (Anagnostaras, Maren, Sage, Goodrich, & Fanselow, 1999; Wallenstein & Vago, 2001) and spatial discrimination task (Carli, Luschi, & Samanin, 1997). Deiana et al.’s comprehensive review (Deiana, Platt, & Riedel, 2011) documents the robust impairments by scopolamine on spatial learning tasks (i.e., typically hippocampus-dependent) when the drug is given prior to the learning event.

Indeed, scopolamine may particularly affect encoding rather than retrieval and consolidation, in line with the ERS framework. For instance, in a spatial maze and a contextual fear conditioning task, scopolamine impaired encoding but not retrieval/consolidation (Rogers & Kesner, 2003,; Rogers & Kesner, 2004), while physostigmine, which elevates ACh levels, had the opposite effect. In humans, scopolamine impaired acquisition but not retrieval of a verbal paired associate task (Atri et al., 2004) that may be hippocampal dependent. Furthermore, exactly in line with the ERS framework’s predictions regarding interference, the muscarinic antagonism disrupted more strongly the acquisition of overlapping word pairs (e.g., leather-holster, then leather-boot), compared to non-overlapping pairs (e.g., leather-holster, then mire-ore). Further work on a verbal paired-associate task showed that cholinergic antagonism impaired encoding, but preserved or even improved consolidation, while cholinergic enhancement impaired consolidation (Gais & Born, 2004; Rasch, Born, & Gais, 2006).

### 2.9 Encoding, retrieval, and consolidation are scheduled by changes in oscillatory activity

While the ERS theorised role for cholinergic transmission is certainly consistent with experimental tests, ACh action can be quite long-lasting (e.g., presynaptic inhibition lasting for up to ∼20 s). Yet, some tasks require a more rapid cycling between encoding and retrieval and consolidation. Thus, it has been proposed that this cycling may be controlled by changes in oscillatory state (Buzsaki, 1989; Hasselmo et al., 2002). We have already noted above that the brain schedules consolidation during sharp wave/ripple states. Cycling between theta (online) and sharp wave/ripple states (offline) thus may resemble cycling between an online state for encoding and retrieval and an offline state for consolidation. Thus, (Hasselmo, 2012; Hasselmo et al., 2002) posited that encoding and retrieval preferentially take place at different phases of the theta cycle in the hippocampus.

### 2.10 Encoding and retrieval occur at different theta phases: The model

**Fig. 2:**
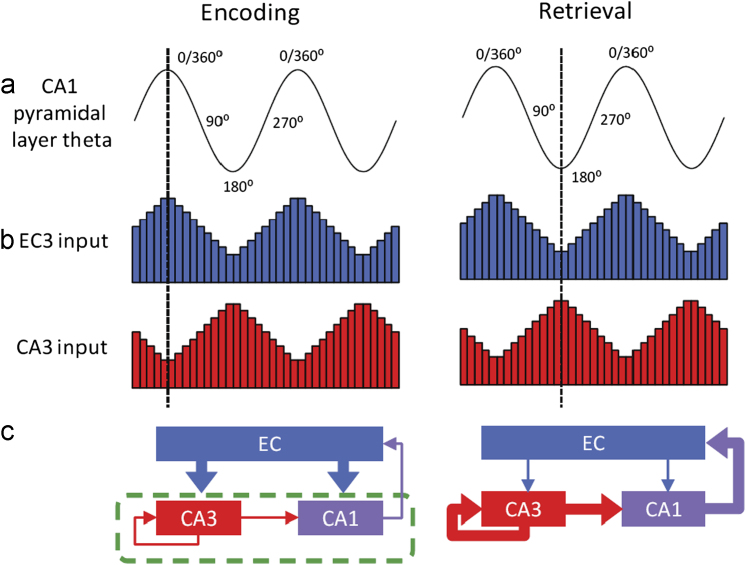
Separation of encoding and retrieval by the phase of theta. *Left column:* (a) Encoding takes place at the peak of CA1 pyramidal layer theta. (b) At this theta phase (vertical dashed line), the synaptic transmission from entorhinal cortex layer 3 (EC3) to CA1 is strong, while transmission from CA3 to CA1 is weak. (c) The thickness of the arrows indicates the strength of excitatory synaptic transmission, with thick (thin) arrows representing strong (weak) synaptic transmission. This differential strength of the EC3 and CA3 synaptic transmission to CA1 allows the EC3 input patterns to be protected against interference from previously learned associations provided by CA3 input. The entorhinal input drives encoding of novel associations in the CA3–CA1 synapses, which undergo long-term potentiation, because they are active at the peak of theta. *Right column:* (a) Retrieval occurs at the trough of CA1 pyramidal layer theta. (b) and (c) At this phase, synaptic transmission from EC3 is weak (but sufficient to cue retrieval), while transmission from CA3 is strong. This permits efficient retrieval of previously learned associations. No encoding involving the retrieved patterns takes place in the CA3–CA1 synapses because synapses active at the theta trough phase do not undergo long-term potentiation or undergo long-term depression. *Green dashed frame* indicates site of action of ACh presynaptic inhibition effects. ACh mediates transitions between encoding and retrieval on a longer timescale than theta cycle transitions by suppressing hippocampal intrinsic connectivity (see Fig. 1). High cholinergic levels in CA1 favour encoding by reducing selectively the intrinsic input from CA3, while relatively sparing the extrinsic input from EC3. In sum, encoding and retrieval dynamics are set by both theta phase and acetylcholine. Adapted from Hasselmo et al. (2012).

Hasselmo (2012) details the model and evidence for it. Here, we update empirical support for the model using recent data from our laboratory. Fig. 2 outlines the idea. As with the ACh-based ERS model presented above in Sections 2.3–2.7, we focus on region CA1 and its two main input regions: the entorhinal cortex (layer 3) and CA3. As Fig. 2a and b indicates, encoding takes place preferentially around the peak of theta as recorded from the CA1 pyramidal layer (left column) when entorhinal cells are maximally active, and CA3 cells are minimally active. Importantly, LTP occurs at the peak of theta not only for the abundantly active sensory afferent entorhinal–CA1 synapses but also for the fewer synapses that are active in CA3–CA3 and CA3–CA1 pathways. In contrast, retrieval takes place preferentially around the trough of theta (right column) when entorhinal cells are minimally active (though sufficiently so to cue retrieval), and CA3 cells are maximally active. The fact that CA3–CA3 and CA3–CA1 transmission is maximally active at this phase means that CA1 activity is dominated by the retrieval of previous associations, such as in pattern completion. Interference between these previous associations with the encoding of new associations is minimised by the absence of LTP of active synapses at this trough phase of theta. Indeed in the case of Hasselmo et al. (2002) instantiation of this model, long-term depression occurs at this phase (Hyman, Wyble, Goyal, Rossi, & Hasselmo, 2003), and consequently, retrieval-induced forgetting (an idea built upon by Norman, Newman, and Detre (2007)). Fig. 2c depicts the synaptic pathways that wax and wane with every theta cycle, with the dashed box showing the pathways subject to ACh’s presynaptic inhibition effects. As with the more slowly transitioning Acetylcholine ERS model, this model minimises interference between the retrieval of previous associations and the encoding of new associations. The network successfully learns a task where a stimulus takes on more than one association: for example the model copes well with spatial reversal learning on T-maze, where a location is initially rewarded, but then is later not rewarded (Hasselmo et al., 2002).

### 2.11 Theta and plasticity and memory

One of the empirical foundations of Hasselmo et al. (2002) model is the strong relationship between theta phase and plasticity. In CA1, LTP at Schaffer collateral (i.e., CA3 to CA1) synapses is preferentially induced by stimulation at the peak of local theta, while stimulation at the trough does not induce LTP and can induce LTD or depotentiation (Hölscher, Anwyl, & Rowan, 1997; Huerta & Lisman, 1993; Huerta & Lisman, 1995,1996; Hyman et al., 2003).

More general empirical bases for the model come from the strong relationship between theta and memory. In humans, the degree of theta-phase locking of individual hippocampal neurons to local theta at the encoding stage of picture presentation predicts the degree to which those pictures are subsequently remembered (Rutishauser, Ross, Mamelak, & Schuman, 2010). EEG and MEG studies (reviewed in Düzel, Penny, and Burgess (2010), Fell and Axmacher (2011)) show that increased theta power and coherence are often associated with the success of encoding and retrieval in humans (e.g., Fell et al., 2003; Guderian & Duzel, 2005; Jacobs, Hwang, Curran, & Kahana, 2006; Klimesch, 1999; Osipova et al., 2006; Sederberg, Kahana, Howard, Donner, & Madsen, 2003). The power of hippocampal theta before stimulus onset can predict encoding success (Guderian, Schott, Richardson-Klavehn, & Duzel, 2009). Conscious recollection, an apparently multi-modal, multi-regional form of retrieval, might be expected to require the coordination of spatially distributed regions by theta oscillations, and is accompanied by an increase in theta power in medial temporal lobe, prefrontal and visual areas (Barbeau et al., 2005; Guderian & Duzel, 2005; Klimesch et al., 2001). In conclusion, theta oscillations appear to be involved in both memory encoding and retrieval.

### 2.12 Evidence that encoding and retrieval take place at different theta phases: Phase of peak spiking in CA3, entorhinal cortex, and CA1

**Fig. 3:**
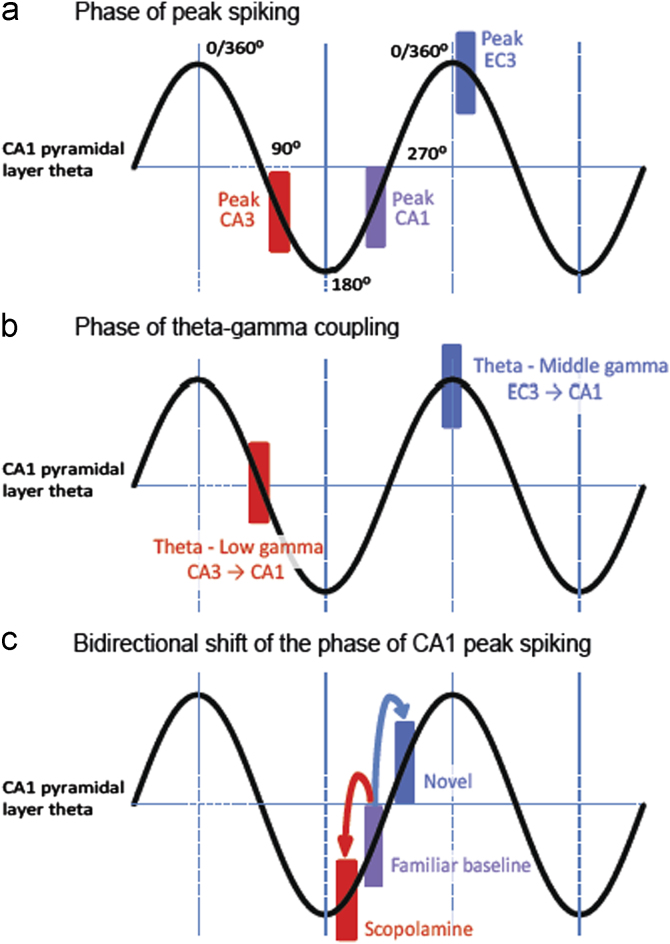
Supporting evidence for the encoding versus retrieval scheduling by theta phase and acetylcholine. (a) Maximal firing of pyramidal cells in CA3 and EC3 occurs around the trough and the peak of CA1 pyramidal layer theta, respectively. Theta obtained from rats running in a familiar environment. The peak of CA1 pyramidal cells firing is after the trough, at a phase approximately intermediate between the CA3 and EC3 peak firing. (b) Theta–gamma coupling in CA1 is modulated by the phase of theta. The strongest theta and middle-gamma coupling (EC3–CA1 communication) is observed at the peak of CA1 pyramidal layer theta, coincident with the phase at which EC3 cell firing is maximal (see (a**)**) above). The coupling between theta and the low gamma is maximal in the descending phase of theta (exact value shown is provisional), potentially close to the peak firing in CA3. (c) Bidirectional modulation of CA1 pyramidal cells main theta phase of firing by novelty and scopolamine during a foraging task. In a highly familiar environment, the main theta phase of firing is slightly after the trough of CA1 pyramidal layer theta (consistent with (a)). In a novel environment, when encoding is expected to prevail, the mean phase of firing is later, closer to the pyramidal layer theta peak and to the phase of EC3–CA1 communication. In novelty, CA1 activity reflects a greater driving by its EC3 inputs, consistent with encoding novel environmental information. Scopolamine (an amnestic cholinergic antagonist), when systemically injected in the familiar environment, induces an opposite effect to that of novelty: the preferred theta phase of firing is now *earlier*, towards the theta trough. This shift towards the theta trough likely reflects a greater driving by CA3, and thus a bias towards retrieval and pattern completion. Scopolamine blocks the shift to a later phase normally elicited by environmental novelty (not shown), in line with the encoding impairment induced by this drug. Parts (a)–(c) based on data as follows: (a) Mizuseki et al. (2009), (b) Colgin et al. (2009), and Scheffer-Teixeira et al. (2011), (c) Douchamps et al. (2011).

For various reasons, few studies have accurately measured the peak phase of spiking in CA1 and its two input regions, the entorhinal cortex (layer 3) and CA3, with reference to CA1 pyramidal layer theta. Recent studies using high volume silicon probe recordings essentially confirm the assumptions of Hasselmo et al. (2002) model (Mizuseki, Diba, Pastalkova, & Buzsáki, 2011; Mizuseki, Sirota, Pastalkova, & Buzsáki, 2009). Encoding-oriented entorhinal layer 3 activity is expected to peak at the peak of theta, and does so; retrieval-oriented CA3 activity is expected to peak at the trough of theta, and peaks on the descending phase towards the trough. Fig. 3a summarises these results showing the peak phase of firing of all 3 regions with respect to the theta recorded from the pyramidal layer of CA1. The peak phase of CA1 spiking occurs after the theta trough, suggesting a value broadly intermediate between the peak phases of its input regions (Ento 3 and CA3).

### 2.13 Evidence that encoding and retrieval take place at different theta phases: Theta–gamma coupling in CA1

The phase of peak spiking activity in a given region is not necessarily identical with the phase of effective communication between that region and a target region. Oscillatory coupling provides a useful clue to effective communication between regions. Recent work, building upon a seminal study by Colgin et al. (2009) has looked at theta–gamma coupling in CA1, and is consistent with the picture described above, assuming that encoding is associated with entorhinal–CA1 activity and retrieval with CA3–CA1 activity. We summarise the evidence here.

Scheffer-Teixeira et al. (2011) examined phase-amplitude coupling between theta phase and the amplitude of different gamma bands in CA1. Phase-amplitude coupling refers to the amplitude modulation of a higher frequency oscillation by a lower frequency oscillation. This work identified two separate gamma frequency bands in the ‘high’ gamma range, one centred ∼80 Hz (here called ‘middle gamma’), and one centred ∼140 Hz (‘high gamma’). Activity in both these gamma bands was controlled by theta phase. Using electrodes located at different depths in CA1, Scheffer-Teixeira and colleagues showed that the strength of the theta-middle-gamma coupling appeared to peak in the lacunosum-moleculare layer, which is the layer where entorhinal axonal terminals synapse onto CA1 dendrites. In other words, theta-middle-gamma coupling probably reflects a state of enhanced communication between entorhinal–CA1 projection neurons and their CA1 targets. So the natural questions are: at what theta phases do the theta-gamma couplings occur that reflect, on the one hand, enhanced entorhinal–CA1 communication (middle gamma), and on the other CA3–CA1 communication (low gamma: ∼40 Hz)? Phase-amplitude coupling data indicates that theta-middle-gamma coupling peaks at the positive peak of CA1 pyramidal layer theta (Scheffer-Teixeira et al., 2011), exactly as predicted by the peak phase of firing in entorhinal layer 3 cells. Complementing this idea is the evidence from low gamma reflecting CA3–CA1 communication. Scheffer-Teixeira et al.’s phase-amplitude study was unable to detect significant theta phase modulation of low gamma activity, but Colgin et al. (2009) found that the preferred phase of theta-low-gamma coupling was on the descending phase of pyramidal-layer theta.

In summary, the evidence from peak phase of spiking in CA1’s input regions and theta–gamma coupling are in register with the model and plasticity data. Encoding-oriented entorhinal layer 3 communication with CA1 at stratum lacunosum-moleculare synapses peaks at the peak of theta, the phase most propitious for LTP. Those recurrent synapses which are active will also be potentiated at this phase. Retrieval-oriented CA3 communication with CA1 at stratum radiatum synapses peaks somewhat before the trough of theta, the phase least propitious for LTP.

### 2.14 Evidence that encoding and retrieval take place at different theta phases: CA1 mean phase is closer to the entorhinal–CA1 transmission peak in novelty, and closer to the CA3–CA1 transmission peak under scopolamine

Another test of the idea that encoding and retrieval in CA1 take place at different theta phases is to compare the theta phase of firing of CA1 pyramidal cells across two conditions: one, when an environmental context is highly familiar, the other, when an environmental context is novel. The assumption is that the balance between encoding and retrieval is biased towards encoding when a context is novel. Over the course of a trial, CA1 activity is expected to be a mixture of both encoding and retrieval, with the encoding-retrieval balance represented by the mean theta-phase of CA1 firing. The clear prediction is that the mean phase will be different in the familiar and novel contexts: closer to the entorhinal–CA1 encoding-oriented phase in novelty, and closer to the CA3–CA1 retrieval-oriented phase in familiarity. We confirmed this prediction (Lever et al., 2010), where we found that the mean phase of firing in CA1 place cells (pyramidal cells) occurs at a later theta phase in a novel environmental context compared to a familiar one. We call this the ‘later-theta-phase-in-novelty effect’. We subsequently manipulated both novelty and cholinergic transmission (Douchamps et al., 2011). As noted above, ACh levels are high under novelty, and we reasoned that ACh is involved in the later-theta-phase-in-novelty effect. Therefore, systemic injection of scopolamine was used to disrupt hippocampal processing, and in particular to examine encoding in tasks which compare encoding to retrieval and/or consolidation.

The results of Douchamps et al. (2011) were clear. Without cholinergic disruption, we replicated the later-theta-phase-in-novelty effect when the environmental context was novel. When scopolamine was given in a familiar environment, scopolamine induced an *earlier* theta phase of firing relative to baseline trials in that environment. Thirdly, when systemic scopolamine was given in the novel environmental context condition, the ‘later-theta-phase-in-novelty effect’ was abolished. Accordingly, there was a bidirectional shift in the mean theta phase of firing in CA1 relative to baseline. A schematic diagram of the results of Douchamps et al. (2011) is shown in Fig. 3c, which depicts the mean phase results with reference to CA1 pyramidal layer theta. Baseline mean phase in CA1 occurs after the pyramidal layer theta trough and shifts closer to the theta peak in novelty and closer to the theta trough under scopolamine. Taken together with the evidence summarised in Fig. 3a and b, Douchamps et al. (2011) study shows that under novelty, when ACh levels are high, the mean phase of spiking in CA1 pyramidal cells reflects a stronger contribution of entorhinal-driven activity, consistent with higher levels of encoding. However, under scopolamine, the mean phase reflects a stronger contribution of CA3-driven activity, consistent with higher levels of retrieval.

In our view, these data support a combination of the two fundaments of the ERS framework, that is, that encoding and retrieval preferentially take place at different phases of hippocampal theta (see also Manns, Zilli, Ong, Hasselmo, & Eichenbaum, 2007), and that ACh biases the system towards encoding. Interestingly, our data indicate that cholinergic muscarinic disruption by scopolamine does not only prevent the appearance of the later-theta-phase-in-novelty effect; scopolamine in the familiar environment produces an earlier phase of firing that takes the mean phase closer to the pyramidal layer theta trough. These results strongly suggest a bias towards retrieval and pattern completion during muscarinic blockade. The probability of new encoding would be greatly reduced.

These pharmacologically-derived data are consistent with the findings of Ikonen, McMahan, Gallagher, Eichenbaum and tanila (2002), who examined the effects of lesions of cholinergic septohippocampal neurons upon place cells in rats exposed to similar-but-different environmental contexts. They found that while cholinergic-lesioned rats’ place cells initially showed pattern divergence (known as ‘remapping’; Bostock, Muller, & Kubie, 1991; Leutgeb, Leutgeb, Treves, Moser, & Moser, 2004; Lever, Wills, Cacucci, Burgess, & O’Keefe, 2002; Muller & Kubie, 1987; Wills et al., 2005) upon exposure to a novel similar-but-different environmental context, the firing patterns in the novel context gradually became more like the patterns in the familiar context. In other words, relative to the unlesioned animals, the dynamics in cholinergic-lesioned animals was biased towards pattern completion.

## 3 Do lesions of the cholinergic system support the model?

It should be noted that much of the work discussed to this point is reliant on the interpretation of pharmacological manipulations of the muscarinic cholinergic system. If the cholinergic system is truly critical for these processes, however, one would expect significant impairment from lesions of the cholinergic input to the hippocampus. Lesions of the fornix (which carry cholinergic projections to the hippocampus) or electrolytic lesions of the medial septum (the region of the basal forebrain from which cholinergic projections to the hippocampus arise) impair a range of hippocampal-dependent tasks (e.g., Kelsey & Landry, 1988; Markowska, Olton, Murray, & Gaffan, 1989; Mitchell, Rawlins, Steward, & Olton, 1982; Nilsson, Shapiro, Gage, Olton, & Bjorklund, 1987). Clearly, lesions of the fornix and electrolytic lesions of the medial septum will result in damage to cells other than those that express ACh, and therefore, the memory impairments are not necessarily an indication of a role for ACh in hippocampal-dependent memory. Specific immunotoxic lesions of the cholinergic projections from the medial septum to the hippocampus can therefore be made, but these lesions often fail to produce impairments in hippocampal-dependent memory tasks (e.g., Baxter et al., 1996; Frick, Kim, & Baxter, 2004; McMahan, Sobel, & Baxter, 1997). Such evidence would suggest that there is no specific requirement for ACh in hippocampal dependent memory (for review see Parent & Baxter, 2004).

These selective lesions of cholinergic projections to the hippocampus are difficult to interpret, however. More widespread lesions of the cholinergic basal forebrain do impair memory in rats, including memory in hippocampal-dependent tasks (e.g., Berger-Sweeney et al., 1994; Leanza, Nilsson, Wiley, & Bjorklund, 1995; Nilsson et al., 1992). These studies often involve intraventricular injections of the immunotoxin which results in damage to cholinergic cells throughout the basal forebrain. However, such non-localised lesions also lead to other deficits, such as motor impairments following damage to cells in the cerebellum (e.g., Waite et al., 1995; Waite, Wardlow, & Power, 1999) which might explain the impaired performance on memory tasks. However, there are some selective immunotoxic lesions of cholinergic cells in MS/VDB which do result in learning and memory deficits (e.g., Lehmann, Grottick, Cassel, & Higgins, 2003; Shen, Barnes, Wenk, & McNaughton, 1996) although it has been argued that in many of these cases selectivity of the lesion to cholinergic cells alone has not been demonstrated, leaving open the option that non-cholinergic mechanisms underlie the impairments (Parent & Baxter, 2004).

Thus, there is a discrepancy between the effects of pharmacological manipulations of ACh in the hippocampus (discussed above) and complete deafferentation of the hippocampus through lesions. However, there are multiple possible reasons for such apparent discrepancies. First, it is possible that following lesions of the cholinergic input to the hippocampus, some compensatory mechanism occurs that allows normal function to occur in the absence of cholinergic input. Such a mechanism might take some time to develop and so would not be invoked by a temporary pharmacological manipulation of the cholinergic system, and therefore, the true effect of ACh in the hippocampus might best be shown by pharmacological manipulations where such compensatory mechanisms do not occur. Second, it is possible that pharmacological manipulations have a more severe impairment on hippocampal-dependent memory because it produces a malfunctioning system rather than removing the system completely. For example, for a given hippocampal-dependent task there might be multiple non-hippocampal possible solutions. In this case lesions of the cholinergic input to the hippocampus would remove the ability to use the hippocampal-dependent strategy and therefore, the animals would switch instead to a non-hippocampal strategy to successfully complete the task. Instead, when pharmacological manipulations are used the animals may still attempt to use the hippocampal-strategy but fail because the strategy is dysfunctional as a result of the cholinergic manipulation.

While this second explanation may initially appear viable, it predicts that the same adaptation to non-hippocampal strategies would be adopted for complete lesions of the hippocampus rather than just cholinergic lesions of the hippocampus. This clearly is not the case, and so it seems unlikely that (for example) non-hippocampal solutions to tasks are adopted only in the presence of a cholinergic depleted hippocampus, but not in the complete absence of a hippocampus. However, the mechanisms discussed earlier suggest that there may be cholinergic and non-cholinergic dependent mechanisms within the hippocampus and that in the absence of cholinergic input (but not in the presence of pharmacological manipulation of the system) animals adapt to a non-cholinergic, but still hippocampal-dependent, strategy. Below we outline the way in which the mechanisms described earlier can be applied to those tasks that are impaired and those tasks that are spared by cholinergic lesions of the MS/VDB.

### 3.1 Explaining the effects of septohippocampal cholinergic lesions

We use the ERS framework to offer an alternative to Baxter et al. (1995) interpretation of their own study. In this study, performance on the standard Morris watermaze task, the place task, was unaffected by lesions of the cholinergic projections to the hippocampus, while the same group’s performance on a delayed-match-to-place version (DMP) of the task was impaired. In the original report, and in Parent and Baxter (2004) review, the deficit is described as likely non-nmemonic. However, it seems possible that the variable delays in the DMP task and the lack of a delay-dependent deficit in the cholinergic-lesioned group obscures the crucial difference between the place task and DMP task. In the place task, which was run first, the goal location was stable across 24 trials over 8 day, while in the DMTP task the goal location changed every day for 16 day (2 trials/day). Thus, the DMP task was a serial reversal learning task, and therefore, the ERS framework would predict an advantage with ACh in this task. On any given day, there is scope for interference, most notably from the retrieval of the previous day’s now incorrect location-platform association, which ACh would help to suppress at the time of encoding the new goal location. The strong prediction is that the cholinergic-lesioned animals’ search path would be biased towards the previous goal location.

Baxter and colleagues commented that the deficit pattern is ‘difficult to interpret as a memory impairment, which would be expected to emerge with increasing delays’ (p.720, Baxter et al., 1995). However, as they acknowledge, like the cholinergic-lesioned animals, the performance of control animals did *not* worsen with delay in the DMP task. Moreover, an impairment in inhibiting associative retrieval during the first ‘encoding’ trial of each day would impair subsequent search across all delays, and it is not obvious that the inhibition of associative retrieval during encoding is a non-mnemonic operation, though it does share with attention the idea of promoting one kind of input at the expense of another. Attentional processing and ERS both share a mechanism of context-specific selection of input streams. Certainly, the ERS framework invites us to view memory operations in terms beyond that of synaptic potentiation/depression and decay.

One other task which has been reliably impaired following lesions of the cholinergic input to the hippocampus is contextual-spatial conditional discrimination learning (Janisiewicz, Jackson, Firoz, & Baxter, 2004). In this task, rats were taught to respond to a location in a spatial array where the correct location was conditional on the context in which the array was presented. The contexts were defined by the shape of the environment and differed in distinctive visual cues on the walls. The spatial array on the floor remained the same in both contexts, merely the location of food within the array changed. In this task, animals with cholinergic lesions of the portion of the basal forebrain which contains the hippocampally-projecting neurons (i.e., medial septum and vertical limb of the diagonal band: MS/VDB) were significantly impaired when both contexts were presented as novel environments at the start of learning, supporting evidence from marmosets that this type of learning is dependent on cholinergic innervations of the hippocampus (Ridley et al., 1999). The finding cannot be simply ascribed to the cholinergic input to the hippocampus being important for applying the conditional rule, as the rule can be learnt and applied in animals with cholinergic lesions of the MS/VDB if one of the contexts is pre-exposed before the conditional learning task (Janisiewicz et al., 2004). Indeed, once again this finding fits with the mechanisms outlined earlier. In the version of the task that is impaired, animals are faced with a large amount of interference, where two equally familiar contexts are presented with different (but equally familiar) spatial locations rewarded in these two contexts. As with the water maze task described above, the locations that the animal has to identify and represent as important change from trial to trial, with different locations represented in each context. In contrast, when one of the contexts (but not both) is pre-exposed to the animals so they are highly familiar with it, then the task is unaffected as the familiarity with one context has increased the discriminability of the two contexts and interference is reduced.

**Fig. 4:**
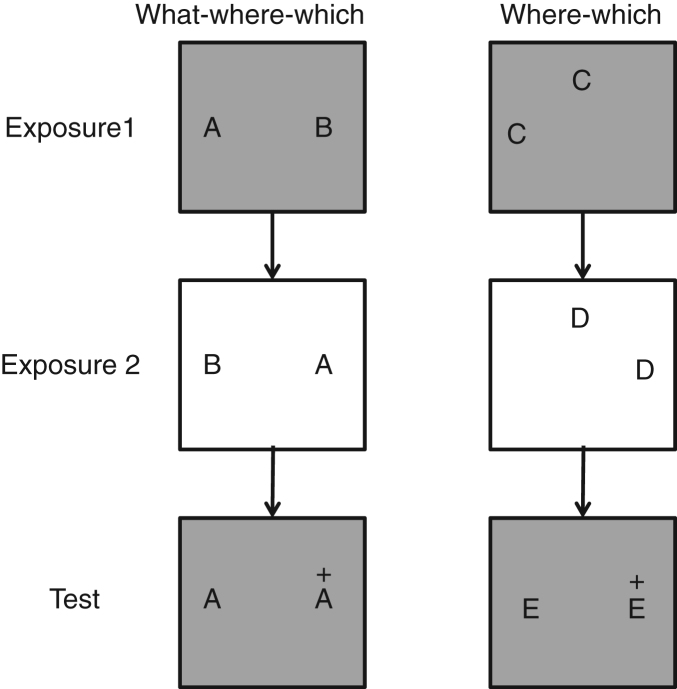
Outline of procedure for what-where-which and where-which tasks. Both the what-where-which (left) and where-which (right) tasks (Easton et al., 2011) involve two exposure events and a later test. The combination of two exposure events and a test comprise a trial, and objects are trial unique (though for simplicity only one set of objects is indicated in the figure). Novel combinations are indicated by ‘+’ and represent configurations of objects, locations and contexts (what-where-which; left) and locations and context (where-which; right) that have not been seen in either exposure event. The trials are counterbalanced between animals on each trial and across trials for each animal. For simplicity only one of the counterbalanced schedules is shown in the figure. Animals are always released in the centre of the arena (1 m×1 m) facing the 12 o’clock position. Each exposure event and test is 2 min long, allowing animals to explore the objects. Exploration at test is recorded and comparative exploration of the novel versus the familiar combination is used as a measure of the animal’s memory.

This context-spatial conditional task has been replicated using a spontaneous behaviour task in rats, rather than tasks that motivate behaviour through food reward. These spontaneous tasks are important in delineating the specific role of regions in particular tasks as animals are unlikely to adopt alternative strategies to solve tasks as the task merely involves exploring objects (Easton & Eacott, in press). Spontaneously, rats preferentially explore novel objects more than familiar ones (Ennaceur & Delacour, 1988). However, if an impairment in memory prevents the rats from identifying one of the objects as familiar the animal is unlikely to adopt a strategy that ensures they can identify one of the objects as more interesting to explore than the other, whereas in food motivated tasks such strategies might be adopted to ensure maximum food intake. In the spontaneous task rats with cholinergic lesions of MS/VDB were impaired at a ‘where-which’ task where an object was found in a location within a context, where no object had been seen in that position in that context previously, thus the occupied location was novel for that context (Easton, Fitchett, Eacott, & Baxter, 2011) (see Fig. 4). This is the spontaneous recognition task equivalent of the context-spatial conditional discrimination task described above (Janisiewicz et al., 2004) and is similarly impaired by cholinergic deinnervation of the hippocampus. However, importantly these animals were also tested on a spontaneous task of episodic memory using novel combinations of ‘what-where-which’ i.e., unique combinations of objects in particular locations against particular contextual environments (Eacott & Norman, 2004) (see Fig. 4). Animals with cholinergic lesions of MS/VDB were unimpaired at this episodic task (Easton et al., 2011).

This finding is particularly surprising for two reasons. First, the episodic task in these rats is based on the scene learning episodic-like task used in monkeys (Gaffan, 1994) which is consistently impaired by cholinergic lesion (Browning et al., 2009; Easton et al., 2002), although the specific role of MS/VDB projections in this task in monkeys has not been assessed. Second, and perhaps more importantly, the what-where-which task requires animals to be able to identify location and context as part of the task (in combination with object information) but location-context information on its own is impaired in the same animals (in the where-which task; Easton et al., 2011). This supports the view that memory for what-where-which is more than merely the sum of its parts, and more likely represents an episodic memory for the specific events that were experienced (Eacott & Easton, 2010; Eacott & Gaffan, 2005; Eacott & Norman, 2004; Easton & Eacott, 2008). However, some component of spatial and contextual memory must be available to the hippocampus (to allow normal performance on the what-where-which task) which is not sufficient to allow normal performance on a task that only requires spatial and contextual information. Although this sounds counterintuitive, some explanation seems possible when differences between these two tasks are considered.

The animals run multiple trials of each type, and each has trial unique objects. However, the contexts and spatial locations of objects within contexts are maintained across trials. Therefore, in the what-where-which task, the locations in which objects are found never changes. On every trial, and on every stage of every trial, objects will be found to the left and right of the animal (which is initially placed in the centre of the arena in the same orientation each trial) even though the objects themselves change location within a trial, and new objects are used for each trial. Although, the same object will not always be in the same location, nonetheless there will always be an object in these locations. In contrast, for the where-which task three locations are used across each trial (although these three locations are the same locations used in every trial). Within a single trial, there is no consistent location where an object (irrespective of specific identity of that object) will be found. For example, on one sample objects will be found at a 9 o’clock and 12 o’clock orientation to the animal’s starting position, whilst on another it may see objects at 12 o’clock and 3 o’clock. In addition, in order to maintain exploration of these locations, the where-which task utilises novel objects on each sample and test phase of every trial. In contrast, in the what-where-which task, objects are trial unique, but (copies of) objects are re-presented in each acquisition phase and at test.

The results of this task are somewhat in contrast to the results of the others described above in that they are less readily explicable by a role of ACh in balancing encoding and retrieval and thereby reducing interference. In both tasks contexts and locations are equally familiar. Indeed, it might appear that there is *more* scope for interference in the what-where-which task as the same objects are used throughout a single trial (although the objects themselves are trial unique) though their location alters in each sampling event and successful performance at test relies on being able to discriminate these highly similar events. Nonetheless, performance is unimpaired by a lesion of the cholinergic projections to the hippocampus. In contrast, the impaired where-which task has more apparent differences between each sample—suggesting that there might be less interference in this task: novel objects are used on all trials and different spatial locations are filled in each context. However, as outlined for the two tasks above, only in the where-which task do locations of interest move between trials (i.e., different locations are filled in different contexts in a single trial) but also within a context across trials. In this way, it is possible that the where-which task actually involves *increased* interference in comparison to the what-where-which task.

One possibility, therefore, is that lesions to the cholinergic input to the hippocampus only impair tasks where salient locations are unstable across or within trials. This is true of Baxter et al. (1995) water maze task, Janisiewicz et al. (2004) context-location conditional discrimination and Easton et al. (2011) where-which task, all of which were impaired by these lesions. It is not true, however, of the what-where-which task that is unimpaired following these lesions (Easton et al., 2011) as although the locations in which individual objects appear change between contexts, the same locations are used in every trial. The mechanisms outlined above might be specifically required for reducing interference when locations matter. Whilst the hippocampus is necessary for normal performance of the what-where-which task (Eacott & Norman, 2004; Langston & Wood, 2010) this may be because the hippocampus is required for combining information about what happened (in this task specifically object information from perirhinal cortex) with information about where and in which context it happened, but the cholinergic input to hippocampus is only necessary for representations of locations and context, irrespective of object identity or indeed the location of particular objects. This possibility is supported by recent evidence that cholinergic input to the hippocampus is necessary for identifying novel salient locations (Cai, Gibbs, & Johnson, 2012) as their spontaneous recognition of novel locations is impaired (whilst their spontaneous recognition of objects is preserved).

## 4 Summary

In summary, we suggest there is good support from pharmacological studies of the ERS framework’s idea that hippocampal ACh controls the balance between encoding and retrieval in that region. Acetylcholine’s range of effects includes promoting the response to novelty by enhancing exploration and synaptic plasticity. Acetylcholine serves to reduce interference in the learning process. Recordings of CA1 pyramidal cells under manipulations of novelty and cholinergic disruption suggest that encoding and retrieval take place at different theta phases, and that this is at least partly controlled by ACh. Acetylcholine enhances those pathways that support the encoding of information inherent in the current context (‘place A’ and ‘food absence’, ‘leather’ and ‘boot’) while inhibiting those pathways subserving retrieval of different associations with some of the stimuli present in the current context (‘place A’ and ‘food presence’, ‘leather’ and ‘holster’). A key prediction of the ERS framework is a particular advantage of cholinergic signalling (i.e., a particular disadvantage following its disruption) in those tasks where a reduction in interference from pre-existing representations can improve performance. Everyday episodic memory typically involves the encoding of novel associations with contexts, locations and cues for which previous associations already exist. The general prediction would be that the degree of potential interference correlates with the impairment from cholinergic disruption. However, interference in hippocampal-based memory may be more problematic with particular modalities of associations. In keeping with the results of lesion studies described above, we propose that the cholinergic input to the hippocampus is especially involved when this reduction in interference relates specifically to locations and contexts, but not to the object identity attached to these representations.

We have noted that one task of episodic memory in rats (Eacott & Norman, 2004) is not impaired by the cholinergic septohippocampal lesions (Easton et al., 2011). We do not conclude that this implies that all episodic memories are spared by cholinergic lesions of the hippocampus; rather, we argue that not all episodic memories are impaired by these lesions. It will be an important research goal to clarify the particular dependencies on cholinergic signalling in particular instances and variants of episodic learning and memory.
